# Endothelial progenitor cells in chronic obstructive pulmonary disease and emphysema

**DOI:** 10.1371/journal.pone.0173446

**Published:** 2017-03-14

**Authors:** Margaret F. Doyle, Russell P. Tracy, Megha A. Parikh, Eric A. Hoffman, Daichi Shimbo, John H. M. Austin, Benjamin M. Smith, Katja Hueper, Jens Vogel-Claussen, Joao Lima, Antoinette Gomes, Karol Watson, Steven Kawut, R. Graham Barr

**Affiliations:** 1 Department of Pathology and Laboratory Medicine, University of Vermont, Burlington, Vermont, United States of America; 2 Department of Medicine, Columbia University, New York, New York, United States of America; 3 Department of Radiology, University of Iowa, Iowa City, Iowa, United States of America; 4 Department of Radiology, College of Physicians and Surgeons, Columbia University, New York, New York, United States of America; 5 Department of Radiology, Hannover Medical School, Hannover, Germany; 6 Department of Cardiology, Johns Hopkins University, Baltimore, Maryland, United States of America; 7 Department of Radiology, UCLA School of Medicine, Los Angeles, California, United States of America; 8 Division of Cardiology, UCLA School of Medicine, Los Angeles, California, United States of America; 9 Penn Cardiovascular Institute Center for Clinical Epidemiology and Biostatistics Pulmonary, Allergy and Critical Care Division, University of Pennsylvania School of Medicine, Philadelphia, Pennsylvania, United States of America; 10 Department of Epidemiology, Mailman School of Public Health, Columbia University, New York, New York, United States of America; The Ohio State University, UNITED STATES

## Abstract

Endothelial injury is implicated in the pathogenesis of COPD and emphysema; however the role of endothelial progenitor cells (EPCs), a marker of endothelial cell repair, and circulating endothelial cells (CECs), a marker of endothelial cell injury, in COPD and its subphenotypes is unresolved. We hypothesized that endothelial progenitor cell populations would be decreased in COPD and emphysema and that circulating endothelial cells would be increased. Associations with other subphenotypes were examined. The Multi-Ethnic Study of Atherosclerosis COPD Study recruited smokers with COPD and controls age 50–79 years without clinical cardiovascular disease. Endothelial progenitor cell populations (CD34+KDR+ and CD34+KDR+CD133+ cells) and circulating endothelial cells (CD45dimCD31+CD146+CD133-) were measured by flow cytometry. COPD was defined by standard spirometric criteria. Emphysema was assessed qualitatively and quantitatively on CT. Full pulmonary function testing and expiratory CTs were measured in a subset. Among 257 participants, both endothelial progenitor cell populations, and particularly CD34+KDR+ endothelial progenitor cells, were reduced in COPD. The CD34+KDR+CD133+ endothelial progenitor cells were associated inversely with emphysema extent. Both endothelial progenitor cell populations were associated inversely with extent of panlobular emphysema and positively with diffusing capacity. Circulating endothelial cells were not significantly altered in COPD but were inversely associated with pulmonary microvascular blood flow on MRI. There was no consistent association of endothelial progenitor cells or circulating endothelial cells with measures of gas trapping. These data provide evidence that endothelial repair is impaired in COPD and suggest that this pathological process is specific to emphysema.

## Introduction

Chronic obstructive pulmonary disease (COPD), the third leading cause of death in the US [1], is defined by airflow limitation that is not fully reversible[2], and is comprised of either chronic bronchitis, emphysema or both. COPD overlaps incompletely with pulmonary emphysema, which is characterized by destruction of the alveolar walls [3].

Endothelial dysfunction has been noted in COPD and particularly emphysema in various contexts [4–8]. Endothelial cells lining the pulmonary microvasculature, in response to injury, are hypothesized to be lost into the circulation due to a “sloughing off” process that releases endothelial microparticles (EMPs) and circulating endothelial cells (CEC). EMPs are reported to be elevated in COPD and emphysema[7,9,10]. No studies have reported on circulating levels of CECs in COPD, of which we are aware, but increased CECs have been observed in diseases of the vascular circulation [11–18]. This process causes the release of a variety of angiogenic factors, most notably, vascular endothelial growth factor (VEGF), which recruits endothelial progenitor cells (EPCs) to the site, presumably to repair the vascular damage [11,19].

The discovery by Asahara et al[20] that EPCs circulate in blood and are capable of becoming mature endothelial cells in culture spurred interest in the role of these circulating cells in disease, particularly cancer [19], cardiovascular disease [20–26] and lung disease [27–31]. Most [6,27,30,32] but not all [33,34] studies in COPD show decreased EPCs compared to controls, although studies to date have been relatively small and the results varied by the markers used to identify EPC populations [35]. Whether the loss of EPCs is due to loss of bone marrow cells, the source of EPCs or due to recruitment of EPCs from the circulation remains unclear.

Furthermore, COPD is a heterogeneous disease and studies on EPCs have not, to date, examined COPD subphenotypes with the exception of one study that showed an association of CD45+CD34+VEGFR2+ cells but not CD45^dim^CD34+ cells with quantitatively defined emphysema[34]. The largely absent examination of EPCs and emphysema is notable, as emphysema, and particularly panlobular emphysema, is classically regarded to have a vascular component [36,37]. Consistent with this thinking, we previously demonstrated that EMPs were elevated in emphysema but were not related to measures of gas trapping suggestive of small airways disease[7].

Much of the work has been complicated by the fact that there is no consensus on the surface markers required to be considered an endothelial progenitor cell, a hematopoietic progenitor cell or a circulating endothelial cell. For EPCs we chose CD34, a general stem cell marker; KDR, also known as VEGFR2, an endothelial cell marker; and CD133, a bone marrow derived stem cell marker [38,39]. For CECs we chose CD45dim to rule out leukocytes, CD146, an endothelial cell marker, CD31, an endothelial cell marker and CD133, to rule out progenitor cells [18]. It is not our goal here to determine the best markers for endothelial progenitor cells or circulating endothelial cells, but rather to define the role of precisely determined cells in a well-characterized sample with varying severity of COPD and matched healthy controls. This work adds to our current knowledge by increasing sample size compared to previous studies, looking at not only COPD but also emphysema and its subtypes, and evaluating two types of endothelial progenitor cells and circulating endothelial cells in the same study.

## Materials and methods

### Study sample

The Multi-Ethnic Study of Atherosclerosis (MESA) COPD Study is a multicenter case-control study nested within two prospective cohort studies, MESA [40] and the Emphysema and Cancer Action Project (EMCAP) [4], in addition to a small number from the community. We selected all eligible participants in the MESA Lung Study [41] and oversampled participants with COPD or emphysema from the remainder of MESA and EMCAP, collected from May 2009 through July 2011. Participants were age 50–79 years with greater than 10 pack-year smoking histories and free of clinical cardiovascular disease, stage IIIb-V kidney disease, asthma prior to age 45, other lung disease, prior lung resection and cancer.

### Ethics statement

Protocols were approved by the institutional review boards of the participating institutions (Columbia University Medical Center, University of Vermont, Johns Hopkins University, Northwestern Medical Center, University of California at Los Angeles, University of Washington, University of Iowa and Hannover University) and the National Heart, Lung and Blood Institute (NHLBI). Written informed consent was obtained from all participants.

### Flow cytometry methods: EPCs and CECs

Freshly drawn whole blood was drawn into heparinized tubes and shipped overnight in temperature controlled packages, as detailed in S1 Supporting Information. Peripheral blood mononuclear cells were prepared and labeled following standardized protocols (see online supplement). We defined EPCs as cells expressing CD34, a stem cell marker, and KDR (also known as VEGFR2), an endothelial cell marker; we further defined less differentiated EPCs (a subset of EPCs) by the additional presence of CD133, a bone marrow derived stem cell marker [42,43]. For CECs, we chose CD45dim to rule out leukocytes, CD146 and CD31 as endothelial cell markers and CD133 to rule out progenitor cells. Samples were fixed in 1% paraformaldehyde and kept refrigerated in the dark until analyzed by flow cytometry (BD LSR II). Endothelial populations were expressed as percent of PBMCs as gated using forward and side scatter and a gating strategy is shown in the online supplement (S3 Fig, S4 Fig). Biovariability and delayed processing impacts were analyzed (S1 Supporting Information, S1 Fig, S2 Fig) and are acceptable for epidemiology studies.

### Spirometry

Spirometry was conducted on a dry-rolling-sealed spirometer (Occupational Marketing, Inc., Houston, TX) in accordance with American Thoracic Society/European Respiratory Society (ATS/ERS) guidelines[44] following the MESA Lung protocol[45].

COPD was defined following the ATS/ERS and GOLD definitions as a post-bronchodilator ratio of the FEV_1_ to the forced vital capacity (FVC) < 0.70 [2,3], and COPD severity was classified as: mild, FEV_1_ ≥ 80% predicted; moderate, 50–80% predicted; and severe, FEV_1_ < 50% predicted [3,20].

### Emphysema on CT

All participants underwent full-lung CTs on 64-slice helical scanners following the MESA-Lung/SPIROMICS protocol (0.984 pitch, 0.5 seconds, 120 kVp) [46]. The milliamperes (mA) were based on body mass index (BMI): 145 for < 20 kg/m2, 180 for 20–30 kg/m2 and 270 for > 30 kg/m2 for participants recruited from MESA and were set at 200 for others.

The extents of emphysema and emphysema subtypes were assessed qualitatively by experienced chest radiologists following a highly standardized protocol without access to other study information, as previously described [47]. The inter-rater intraclass correlation coefficient of extent of emphysema and emphysema subtypes were 0.77 and 0.42–0.93, respectively [47].

The percent of emphysema-like lung (percent emphysema) was defined as the percent of lung voxels below -950 HU at total lung capacity and gas trapping on CT was defined as voxels below -856 HU on expiratory scans in a subset, both assessed using Apollo 1.2 software (Vida Diagnostics)[46].

### Magnetic resonance imaging

Pulmonary microvascular blood flow was assessed on MRI at one site using a modified version of the cardiac MRI protocol of the fifth examination of MESA on a 1.5 Tesla whole-body MR system (Signa LX, GE Healthcare) with phased-array coil for signal reception using a coronal Time Resolved Imaging of Contrast Kinetics (TRICKS) sequence and a contrast bolus of 0.1 mmol/kg bodyweight gadolinium diethylenetriaminepentaacetic acid (Magnevist, Berlex, Wayne, NJ) at an injection rate of 5 mL/s. Mean values of pulmonary microvascular blood flow and volume were assessed on a coronal slice at the level of the trachea in the peripheral 2 cm of the lung, as previously described[48].

### DLco and plethysmography

DLco and plethysmography were performed in participants at the same site. Single-breath DLco was measured with a Sensormedics Autobox 220 Series instrument (Viasys Healthcare, Yorba Linda, CA) following ATS/ERS guidelines[49]. Body plethysmography was performed using a V6200 Series Autobox (Sensormedics, Yorba Linda, CA) following ATS/ERS recommendations [50].

### Covariates

Age, gender, race/ethnicity, educational attainment, smoking status, pack-years, and medical history were self-reported. Medication use was assessed by medication inventory.[51] Height, weight, blood pressure, oxygen saturation, glucose, total cholesterol, high-density lipoprotein (HDL) levels and complete blood counts were measured using standardized approaches. Smoking status was confirmed by cotinine.

### Statistical analysis

EPCs and CECs displayed skewed distributions and were therefore log-transformed. The association between each of the cellular measures and COPD status and severity was tested in linear regression models in which categories of COPD status and severity were treated as independent variables and cellular populations were treated as the dependent variables. A test of trend across categories of COPD severity was performed, in addition to pairwise comparisons.

Linear regression models were then used to adjust for potential confounders, which were selected based on biologic plausibility and correlations with covariates. The base model was adjusted for age, gender, race/ethnicity and cohort of selection. We then additionally adjusted for smoking status and pack-years, then additional potential confounders of educational attainment, diabetes, hypertension, oxygen saturation, sleep apnea, height, BMI, as they are known confounders for COPD, and statin use, HDL, and white blood cell (WBC) count, as statins have been implicated in endothelial progenitor cell mobilization [52–54], HDL may affect endothelial health and is related to percent emphysema [55], and WBCs to account for unrelated inflammatory white cell interference. Models for the qualitatively measured percent emphysema were additionally adjusted for mA. Models for pulmonary perfusion were additionally adjusted for cardiac output.

The associations of cellular measures with emphysema extent and other continuous outcomes were assessed using linear regression. Because the study recruited based upon COPD status, the assumption underlying linear regression of independent observations may not hold. Analyses of continuous outcomes were therefore weighted according to cohort-specific probabilities of selection and enrollment, with cases recruited from the community assigned the same weights as those from EMCAP.

The p-values were two-tailed with statistical significance defined as p<0.05. Analyses were performed in SAS 9.2 (Cary, NC) and R version 2.14.1 (Vienna, Austria).

## Results

**Fig 1:**
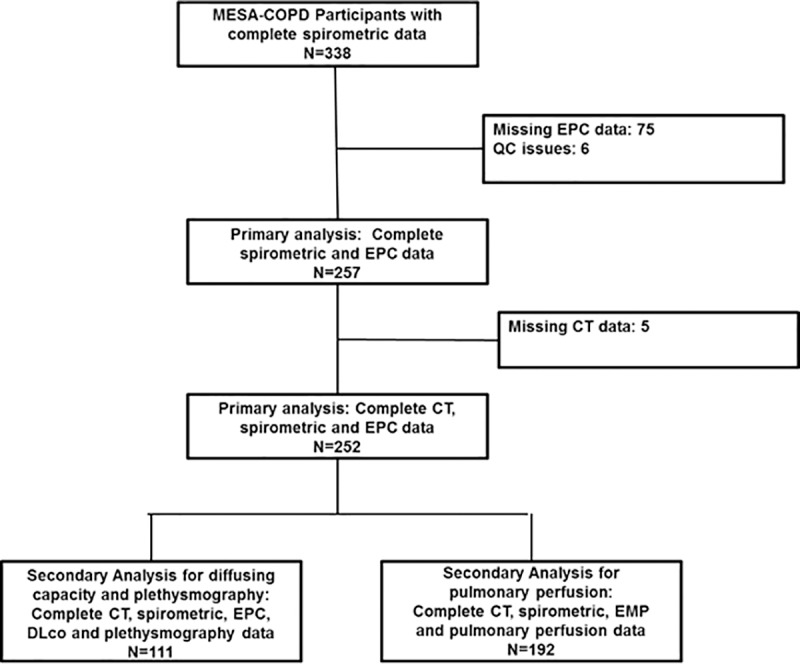
Flowchart of study participants. COPD = chronic obstructive pulmonary disease; CT = computed tomography; EMP = endothelial microparticle; MESA = Multi-Ethnic Study of Atherosclerosis.

The flow diagram for the participants with spirometry, CT and endothelial cell populations is shown in Fig 1. The 257 participants had a mean age of 68.3 +/- 6.9, 28.0% were current smokers, and the race/ethnic distribution was 51.3% white, 36.9% African-American, 14.8% Hispanic and 7.0% Chinese.

Table 1 shows the clinical characteristics of the study sample stratified by COPD severity. Participants with severe COPD were more likely to be male, white and have greater packyears of smoking.

**Table 1:** Clinical characteristics of participants in the MESA COPD Study with measures of endothelial progenitor cells, stratified by COPD severity.

		COPD		
	Controls	Mild	Moderate	Severe/Very Severe
	n = 141	n = 49	n = 54	n = 13
Age, mean (SD), years	69.08 (6.49)	68.78 (7.32)	68.33 (7.62)	67.85 (6.89)
Sex, male, No. (%)	77 (54.61)	36 (73.47)	32 (59.26)	9 (69.23)
Race/ethnicity				
White, No. (%)	62 (43.97)	31 (63.27)	30 (55.56)	9 (69.23)
African American, No. (%)	36 (25.53)	12 (24.49)	17 (12.96)	4 (30.77)
Other, No. (%)	43 (30.5)	6 (12.25)	7 (12.96)	0 (0.0)
Educational Attainment				
High School Degree or Less, No. (%)	37 (26.24)	11 (22.45)	13 (24.07)	2 (15.38)
Some College but no 4yr degree, No. (%)	44 (31.21)	12 (24.49)	10 (18.52)	4 (30.77)
College Degree or higher, No. (%)	60 (42.55)	26 (53.06)	31 (57.41)	7 (43.85)
Height, mean (SD), cm	166.9 (9.41)	171.6 (8.83)	170.10 (9.04)	169.38 (10.24)
Weight, mean (SD). kg	80.71 (18.90)	79.62 (16.41)	79.39 (20.70)	78.82 (18.58)
Body Mass Index, mean (SD), kg/m2	28.86 (5.86)	26.89 (4.25)	27.18 (5.81)	27.35 (5.35)
Cigarette Smoking Status				
Former, No. (%)	109 (77.30)	35 (71.43)	31 (57.41)	10 (76.92)
Current, No. (%)	32 (22.70)	14 (28.57)	23 (42.59)	3 (23.08)
Pack-years of smoking, median (IQR)	28.5 (18.0, 40.5)	33.2 (25.0, 58.0)	39.8 (25.5, 54.7)	40.0 (25.0,70.7)
LDL, mean (SD), mg/dL	111.07 (31.93)	108.35 (31.75)	97.04 (29.85)	106.38 (30.15)
HDL, mean (SD), mg/dL	56.06 (18.21)	57.98 (15.88)	59.02 (20.07)	55.54 (15.45)
Triglycerides, mean (SD), mg/dL	111.63 (52.56)	104.06 (15.90)	110.46 (51.33)	125.31 (67.15)
Cholesterol, mean (SD), mg/dL	189.48 (39.56)	187.08 (36.14)	178.11 (34.07)	186.85 (41.22)
Systolic Blood Pressure, mean (SD), mmHg	122.16 (19.65)	122.59 (15.58)	124.61 (15.29)	128.12 (12.31)
Diastolic Blood Pressure, mean (SD), mmHg	69.28 (9.82)	71.44 (9.73)	72.27 (9.26)	75.96 (9.95)
Hypertension, No. (%)	61 (43.26)	22 (44.90)	25 (46.30)	5 (38.46)
Fasting Glucose, median (IQR), mg/dL	97.0 (91.0,106.0)	98.0 (88.0, 108.0)	100.0 (95.0,109.0)	100.0 (92.0,106.0)
Diabetes Mellitus, No. (%)	23 (16.31)	5 (10.20)	9 (16.67)	3 (23.08)
Statin, No. (%)	50 (35.46)	22 (44.90)	26 (48.15)	4 (30.77)
DLco VA % predicted, mean (SD), n = 118	79.86 (13.14)	70.84 (14.92)	71.33 (19.64)	63.68 (16.74)
RV % predicted, mean (SD), n = 118	68.75 (18.63)	82.94 (18.59)	94.04 (25.57)	138.09 (32.79)
TLC % predicted, mean (SD), n = 118	88.82 (12.48)	99.94 (11.64)	92.55 (12.21)	101.96 (11.06)
RV/TLC ratio, mean (SD), n = 118	0.31 (0.07)	0.31 (0.06)	0.39 (0.08)	0.50 (0.08)
Percent Emphysema_-910_, median (IQR)	14.38 (6.87, 25.50)	25.96 (15.47,35.39)	21.64 (11.52,33.81)	36.82 (32.99,51.48)
Oxygenation Saturation, mean (SD), %	97.29 (1.57)	96.78 (1.87)	96.96 (1.75)	95.75 (2.12)
Home Oxygen Therapy, No. (%)	1 (0.71)	0 (0.0)	1 (1.85)	5 (68.46)
Sleep Apnea, self-reported, No. (%)	10 (7.09)	3 (6.12)	6 (11.11)	3 (23.08)
Cells, median (IQR)				
CD34+KDR+ (%PBMCs [x10^-3^])	111 (48, 266)	74 (43, 231)	90 (40, 148)	38 (33,94)
CD34+KDR+CD133+ (%PBMCs [x10^-3^])	26 (11,55)	18 (8,36)	22 (10,48)	14 (9,28)
CD45dimCD31+CD146+CD133- (%PBMCs)	0.8 (0.3, 2.0)	0.9 (0.5, 2.0)	0.7 (0.4, 1.4)	0.7 (0.3. 1.0)

### EPCs, CECs and COPD severity

The CD34+KDR+ EPC population showed a significant decrease with increasing COPD severity in minimally adjusted (p = 0.03 for trend), smoking adjusted (p = 0.01 for trend) and fully adjusted (p = 0.02 for trend) models (Table 2). Compared to controls, CD34+KDR+ cells were significantly lower in moderate and severe COPD. For CD34+KDR+CD133+ cells, only severe cases were significantly different from controls. There was no evidence that CECs were increased across categories of COPD severity (Table 2).

**Table 2:** Mean difference in endothelial progenitor cells and circulating endothelial cells by COPD severity.

	Controls	Mild	Moderate	Severe	P-trend
	n = 141	n = 49	n = 54	n = 13	
**CD34+KDR+ (as % PBMCs [x10-3])**					
Model 1,† log mean difference	Reference	-0.36	-0.56*	-1.36*	**0.03**
Model 2,‡ log mean difference	Reference	-0.38	-0.60*	-1.51*	**0.01**
Model 3,§ log mean difference	Reference	-0.43	-0.62*	-1.50*	**0.02**
**CD34+KDR+CD133+ (as % PBMCs [x10-3])**					
Model 1,† log mean difference	Reference	-0.20	-0.13	-0.87*	**0.05**
Model 2,‡ log mean difference	Reference	-0.20	-0.11	-0.90	**0.04**
Model 3,§ log mean difference	Reference	-0.26	-0.18	-0.88*	0.07
**CD45dimCD31+CD146+CD133- (as % PBMCs)**					
Model 1,† log mean difference	Reference	0.03	-0.27	-0.48	0.29
Model 2,‡ log mean difference	Reference	-0.03	-0.27	-0.53	0.90
Model 3,§ log mean difference	Reference	-0.06	-0.16	-0.28	0.61

* P-value < 0.05

† Model 1 adjusted for age, gender, race/ethnicity and cohort.

‡ Model 2 adjusted for variables in model 1 in addition to smoking status, and pack-years.

§ Model 3 adjusted for variables in model 2 in addition to educational attainment, body mass index, height, diabetes mellitus, hypertension, oxygen saturation, white blood cell count, sleep apnea, HDL, statin use and high mAs.

### EPCs, CECs and Emphysema

Whereas there was little-to-no evidence that CD34+KDR+ EPCs were reduced with greater extent of emphysema, CD34+KDR+CD133+ EPCs were significantly lower with total emphysema (Table 3).

**Table 3:** Mean differences in endothelial progenitor cells and circulating endothelial cells according to the extent of emphysema and emphysema subtypes on radiologist interpretation.

	Total Emphysema (per log unit increase)		Centrilobular Emphysema (per log unit increase)		Panlobular Emphysema (per log unit increase)		Paraseptal Emphysema (per log unit increase)	
n = 243		p-value		p-value		p-value		p-value
**CD34+KDR+ (as % PBMCs [x10-3])**								
Model 1†	-0.05	0.79	0.03	0.85	-0.39	**0.03**	0.02	0.94
log mean difference	(-0.27, 0.18)		(-0.26, 0.31)		(-0.74, -0.04)		(-034, 0.37)	
Model 2‡	-0.10	0.36	-0.05	0.36	-0.42	**0.02**	-0.03	0.88
log mean difference	(-0.32, 0.12)		(-0.32, 0.22)		(-0.77, -0.06)		(-0.39, 0.33)	
Model 3§	-0.11	0.42	-0.06	0.68	-0.45	**0.03**	0.04	0.84
log mean difference	(-0.36, 0.15)		(-0.34, 0.23)		(-0.86, -0.04)		(-0.39, 0.48)	
**CD34+KDR+CD133+ (as % PBMCs [x10-3])**								
Model 1†	-0.16	**<0.001**	-0.17	**0.04**	-0.32	**0.009**	-0.20	0.25
log mean difference	(-0.32, -0.01)		(-0.33, -0.01)		(-0.56, -0.08)		(-0.53, 0.14)	
Model 2‡	-0.18	**0.03**	-0.19	**0.04**	-0.32	**0.008**	-2.1	0.20
log mean difference	(-0.34, -0.02)		(-0.37, -0.01)		(-0.56, -0.08)		(-0.54, 0.11)	
Model 3§	-0.19	**0.03**	-0.18	0.06	-0.33	**0.009**	-0.25	0.13
log mean difference	(-0.37, -0.02)		(-0.37, 0.01)		(-0.57, -0.08)		(-0.57, 0.07)	
**CEC (CD45**_**dim**_**CD31+CD146+CD133- as % PBMCs)**								
Model 1†	-0.01	0.91	-0.01	0.97	-0.04	0.40	-0.04	0.81
log mean difference	(-0.21, 0.19)		(-0.25,0.24)		(-0.46, 0.18)		(-.036, 0.28)	
Model 2‡	-0.04	0.68	-0.06	0.65	-0.15	0.39	-0.06	0.71
log mean difference	(-0.25, 0.16)		(-0.31, 0.19)		(-0.49, 0.19)		(-0.38, 0.26)	
Model 3§	0.02	0.86	-0.03	0.85	-0.13	0.50	0.14	0.51
log mean difference	(-0.21, 0.25)		(-0.07, 0.24)		(-0.51, 0.25)		(-0.27, 0.54)	

† Model 1 adjusted for age, gender, race/ethnicity and cohort.

‡ Model 2 adjusted for variables in model 1 in addition to smoking status, and pack-years.

§ Model 3 adjusted for variables in model 2 in addition to educational attainment, body mass index, height, diabetes mellitus, hypertension, oxygen saturation, white blood cell count, sleep apnea, HDL, and statin use.

**Fig 2:**
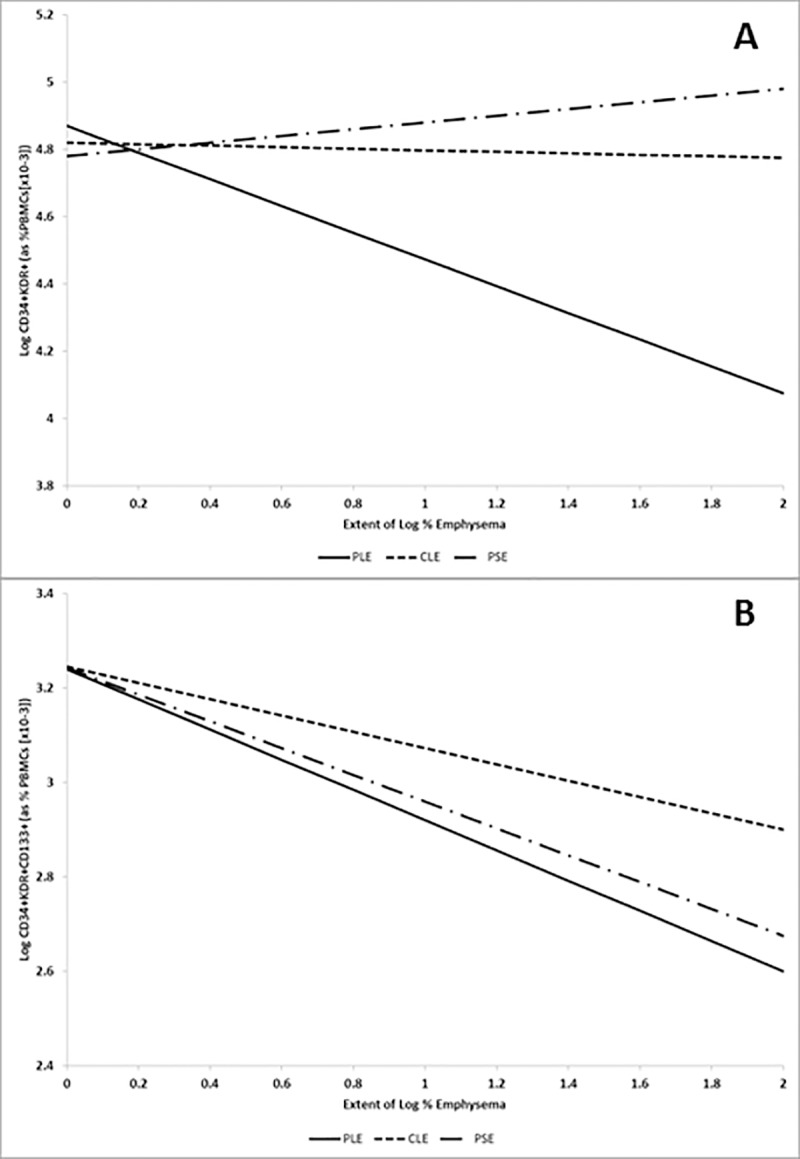
Representation of EPCs levels with extent of emphysema on log-log scale by emphysema subtype. Lines represent modelling results after adjustment for age, gender, race/ethnicity, cohort, smoking status, pack-years, educational attainment, body mass index, height, diabetes mellitus, hypertension, oxygen saturation, white blood cell count, sleep apnea, HDL, and statin use. Panel A: CD34+KDR+. Panel B: CD34+KDR+CD133+. PLE is panlobular emphysema, CLE is centrilobular emphysema and PSE is paraseptal emphysema.

Evaluation by emphysema subtypes demonstrated that CD34+KDR+ and particularly CD34+KDR+CD133+ EPCs were significantly decreased with increasing extent of panlobular emphysema (Table 3). CD34+KDR+CD133+ EPCs only were (weakly) associated with centrilobular emphysema and there was no evidence for associations with paraseptal emphysema. Fig 2 illustrates the association of CD34+KDR+ and CD34+KDR+CD133+ populations with major emphysema subtypes. CEC cells showed no association with any qualitative emphysema measure (Table 3). There was no evidence for an association of either EPC population or CECs with percent emphysema when assessed qualitatively (adjusted P = 0.24, P = 0.92 and P = 0.39, respectively).

### Diffusing capacity and pulmonary microvascular perfusion

Lower levels of CD34+KDR+ and CD34+KDR+CD133+ EPCs were significantly associated with lower diffusion capacity and lower diffusing capacity corrected for alveolar volume in fully adjusted models (S1 Table). CECs were positively associated with diffusion capacity in the fully adjusted model but not in minimally adjusted models or when corrected for alveolar volume.

There were no statistically significant associations between EPCs and pulmonary microvascular blood flow on MRI in fully adjusted models (Online Supplement Table 1). Higher levels of CECs were significantly associated with reduced pulmonary microvascular blood volume in both the minimally and fully adjusted models.

### Gas trapping

None of the endothelial cell populations were associated with increased residual volume (RV) or RV/total lung capacity on plethysmography. Gas trapping on CT was associated with a small but significant decrease in the CD34+KDR+ endothelial progenitor cells but otherwise associations were non-significant (S2 Table).

## Discussion

EPC populations were reduced in COPD, with CD34+KDR+ EPCs in particular being lower in both moderate and severe COPD compared to controls. CD34+KDR+CD133+ EPCs were preferentially lower with greater emphysema on CT and both EPC populations were lower with greater panlobular emphysema and reduced diffusing capacity. CECs were inversely related to pulmonary microvascular blood flow. In contrast, there was little evidence to support a definitive role for endothelial cell populations and measures of gas trapping. These apparent discrepancies in gas trapping results with respect to cellular measures may be explained, in part, by the fact that not all measures of hyperinflation are created equal [56], where gas trapping was associated with smaller airway lumen diameter, greater dyspnea and chronic bronchitis, and hyper-expansion was associated with percent emphysema, lower BMI and higher hemoglobin levels.

To our knowledge, this is the largest study of endothelial cell populations in COPD and the only one to look at more than one EPC population and COPD subphenotypes. The two mechanisms for reduced circulating cell populations are increased utilization, decreased supply or a combination of both. Since the less mature progenitors (CD34+KDR+CD133+) were significantly reduced only in severe COPD, while the CD34+KDR+ cells were highly significant even after adjustment, these findings support the hypothesis that CD34+KDR+ cells were being preferentially drawn from the circulation during mild and moderate COPD (increased utilization) and it is not until severe COPD that the more bone marrow derived cells (CD34+KDR+CD133+) become depleted (decreased supply). Consistent with this thinking, a study by Peinado et al[57] examining lung tissue from persons with COPD and controls showed an increase in CD34+KDR+ cells in the lung tissue of COPD patients. Additionally, EPCs from COPD patients have been shown to be dysfunctional in proliferation and migration assays in response to specific chemotactic factors (i.e. SDF-1α) [58–61], which may be responsible for a change in circulating EPCs. Together, these data suggest that with increasing severity of COPD, CD34^+^KDR^+^ cells are drawn into damaged lung tissue, thus decreasing the number circulating in the peripheral blood. A recent study in mice showed a decrease in circulating CD34+KDR+ cells within 1 day of lps challenge and ~5% of the proliferating pulmonary vascular endothelial cells were of BM-derived cells[62]. They also noted that the resident proliferating vascular endothelial cells were expressing CD34 and KDR on their surface during the reparative process.

Interestingly, a different situation may occur in emphysema, where the extent of emphysema was related to less mature progenitors (CD34+KDR+CD133+) and particularly in panlobular emphysema. These findings suggest that while COPD is recruiting from the circulating CD34+KDR+ EPC pool, emphysema repair relates to the less differentiated CD34+KDR+CD133+ EPCs, the exact mechanism of which remains unclear, emphasizing the differences in this phenotype. The strong association of the EPCs with the panlobular emphysema, which is a more vascular-related emphysema [36,37], is also consistent with recent work showing effects of alpha1 antitrypsin on pulmonary endothelial health [63,64]. This notion is further supported by results for diffusing capacity, which provides an indirect vascular measure, although direct measures of microvascular structure and function on MRI were non-significant for EPCs and only revealed an association for CECs.

In contrast, there was little to no association between endothelial cell populations and measures of gas trapping, which suggests that the biology of endothelial damage and repair may be specific to emphysematous changes in COPD. This data, taken together, illustrates some of the differences in the pathology of COPD and supports further study of sub-phenotypes of COPD and emphysema to better direct future treatment options.

These results suggest that the definition of EPCs and selection of COPD patients and subphenotypes may explain some of the controversy in the literature regarding the role of EPC’s in COPD. Many studies [6,27,35,61,65–67] have used CD34+KDR+ or CD34+KDR+CD133+ cells to study COPD, coming to different conclusions based on the characterization of the EPCs, and few have examined COPD subphenotypes. CD133 has been shown to be a marker of early EPC differentiation that is lost as the progenitor differentiates [68]. Thus, studies that utilize CD133 as a marker are looking at less differentiated EPCs, while those that use just CD34+KDR+ are examining both early and late EPC. Our results suggest that the two cell types behave differently, dependent on the phenotype, suggesting increased uptake of EPCs in COPD but loss of bone-marrow derived cells in panlobular emphysema.

One surprising result was the lack of increase in CECs in COPD or emphysema. We hypothesized that damage to pulmonary vasculature would cause an increase in the “sloughing off” of endothelial cells thus increasing the CEC based, in part, on prior findings for EMPs in this study[7] and the reports of elevated CECs in cardiovascular disease[69]. It is possible these negative results may be due, at least in part, to apoptosis of the endothelial cells in the pulmonary vessels, either on the vessel surface, or shortly after the “sloughing off” process. We and others have previously found evidence for pulmonary vascular endothelial cell apoptosis based on elevated apoptotic EMPs in mild, moderate and severe COPD [7,9,10], consistent with animal[70,71] and human studies implicating apoptosis in emphysema and COPD [72–75]. Further, the study by Boos et al[76] showed that CEC levels correlate with damage markers but not with apoptosis. Additionally, a recent study demonstrating that smoking [77] alters the alpha-1 antitrypsin interaction with caspases causing an increase in endothelial apoptosis supports an apoptotic mechanism in emphysema. And lastly, CECs, as we have defined them, may not be a good indicator of what is happening at the local level.

It was also somewhat unusual that the associations for emphysema were specific to radiologist-defined emphysema and non-significant for quantitatively assessed percent emphysema. The latter null result may have been due to the inability of quantitatively assessed emphysema to define subtypes of emphysema.

The strengths of this study are the precisely defined cellular measures, the relative size of the study and the detailed EPC and COPD subphenotyping. Weaknesses include the delayed sample processing, which we attempted to minimize with strict adherence to timing protocols [78–80] and which is mitigated by the observed correlation between results from fresh and delayed processing among volunteers. Circulating cellular levels may not be reflective of localized events, yet they provide one of the best methods for evaluating cells in living persons. The choice of cellular markers is, to some degree, subjective but was based on best information available at the inception of this project. Finally selection bias can affect case-control studies but this was minimized by using a nested design with known sampling probabilities within MESA and EMCAP.

In conclusion, CD34+KDR+ cells were significantly decreased with increasing COPD severity. The less differentiated EPC phenotype, CD34+KDR+CD133+ cells, was also lower in severe COPD. These results support the hypothesis that the CD34+KDR+ EPC are being preferentially recruited into the lung for vascular repair and the exhaustion of bone marrow derived cells does not appear to occur until spirometry defined COPD has reached severe levels. In contrast, CD34+KDR+ and CD34+KDR+CD133+ cells were reduced in panlobular emphysema and with reduced diffusing capacity, possibly suggesting a loss of bone-marrow-derived EPCs in that form of the disease. This would suggest that the lung vascular injury/repair mechanism for emphysema subtypes varies greatly and requires further evaluation.

## Supporting information

S1 Supporting Information Contains detailed flow cytometry Methods and Assay Validation. (DOCX)

S1 Fig Biovariability. Biovariability data on 13 volunteers, measured over 18 months for CD34+KDR+ EPCs, expressed as # of CD34+KDR+ EPCs per 10,000 lymphocytes.
(TIF)

S2 Fig Plot of freshly processed CD34+KDR+ EPCs versus whole-blood that was stored at controlled room temperature for 24-hours prior to processing and labeling. (TIF)

S3 Fig Representative flow cytometry plots for EPCs. PBMCs were gated based on forward and side scatter properties. A) Forward vs. side scatter to set PBMC gate. B) FITC isotype gate of the gated PBMCs. C) PE Isotype gate of the gated PBMCs. D) APC Isotype gate of the gated PBMCs. E) FITC-CD34+ gated on PBMCs. F) PE-VEGFR2 (KDR)+ gated on the FITC-CD34+cells. G) APC-CD133+ gated on FITC-CD34+PE-VEGFR2(KDR)+ cells. Region F is used as the CD34+KDR+ EPCs and Region G is the CD34+KDR+CD133+ EPCs, both expressed as % PBMCs.
(TIF)

S4 Fig Representative flow cytometry plots for CECs. Gating strategy utilizes both quad statistics and single color histograms to determine gate placement. A) PBMCs are gated using forward and side scatter; B) PeCy5.5 Isotype gate of the gated PBMCs; C) FITC isotype gate of the gated PBMCs; D) PE Isotype of the gated PBMCs; E) APC isotype of the gated PBMCs; F) PE-Cy5.5 CD45dim gate of the gated PBMCs; G) FITC-CD146+ of the PE-Cy5.5CD45dim gate; H) PE-CD31+ of the PE-Cy5.5 CD45dim FITC-CD146+ cells; I) CD133- of the PE-Cy5.5 CD45dim FITC-CD146+PE-CD31+ cells (This CD133- population is the CECs); J) Plot of PE-CD31 vs FITC-CD146 of the gated PBMCs using quad stats; K) PeCY5.5CD45dim vs APC-CD133 plot of the PE-CD31+FITC-CD146+ cells (The PE-Cy5.5CD45dimAPC-CD133- population is the CECs).
(PDF)

S1 Table Mean differences in endothelial progenitor cells and circulating endothelial cells according to related to pulmonary perfusion on MRI and diffusing capacity (DOCX)

S2 Table Association between EPCs, CECs and air trapping. (DOCX)
