# Supplementary material for: Endothelial progenitor cells in chronic obstructive pulmonary disease and emphysema
Source: PLoS One. 2017 Mar 14;12(3):e0173446. doi: 10.1371/journal.pone.0173446 (PMC5349667; doi:10.1371/journal.pone.0173446)
Supplement: S1 Supporting Information — (DOCX) [file pone.0173446.s001.docx]

**Flow Cytometry Methods: EPCs and CECs**

Venous blood samples were drawn into 10 ml heparin tubes after participants sat quietly for 10 minutes following a 12-hour fast. The heparin blood draw tube was inverted gently to ensure proper mixing and maintained at room temperature until packaged according to IATA protocols using containers and packing materials (SafTPak Cat#STP-308) that maintain internal temperature at 15-30^o^C for overnight shipping to a central laboratory. Upon arrival at the central laboratory, samples were placed on a rocker for 10 minutes then centrifuged (1500 x g, 20 minutes, no brake, 25^o^C). Plasma was removed. Peripheral blood mononuclear cells (PBMCs) were prepared from the remaining cell layer using Histopaque (Sigma cat#H10771) [[1](#_ENREF_4)]. The PBMCs were washed and adjusted to 1x10^7^cells/ml.

We defined EPCs as cells expressing CD34, a hematopoietic stem cell marker, and KDR (also known as VEGFR2), an endothelial cell marker; we further defined less differentiated EPCs by the additional presence of CD133, a bone marrow derived hematopoietic stem cell marker [[2](#_ENREF_5), [3](#_ENREF_6)]. For CECs, we chose CD45 to rule out leukocytes, CD146 and CD31 as endothelial cell markers and CD133 to rule out progenitor cells. EPCs were labeled with FITC-anti-CD34(BD #555821), PE-anti-KDR (R&D# FAG357P), and APC-anti-CD133 (Miltenyi #130-90-826). CECs (10^6^ PBMC cells) were labeled with FITC-anti-CD146 (Miltenyi #130-092-851), PE-anti-CD31 (BD #555446), and APC-anti-CD133 (Miltenyi #130-090-826). An appropriate isotype control tube containing 10^6^ PBMC cells was also prepared. Cells were incubated for 20 minutes at room temperature, red cells were lysed with PharmaLyse (BD #555899), and samples were fixed in 1% paraformaldehyde. Samples were kept refrigerated in the dark until analyzed by flow cytometry (BD LSR II). Compensation was performed with single color controls and negative gates were set with appropriate isotype controls. The flow cytometer was set to collect 500,000 events. Endothelial populations were expressed as percent of PBMCs as gated using forward and side scatter. Representative flow cytometry plots are shown in S3 Fig (EPC) and S4 Fig (CEC).

To determine the potential usefulness of EPC measurements in an epidemiology setting, 13 local volunteers were drawn repeatedly over an 18 month period and their EPC levels assessed. As seen in S1 Fig, a large degree of variability within subjects can be seen, but differences between people are also evident. We performed a nested analysis of variance[[4](#_ENREF_7)] to determine analytic (CVa), within subject (CVi) and with group (CVg) coefficients of variation, so that an Index of Individuality (II) can be determined. The index of individuality (CVi/CVg) for EPC = 0.71, which indicates a useful measurement in epidemiology settings where II<1.0 have proven effective for biomarkers[[5](#_ENREF_8)].

The potential impact of the delayed processing due to transport to the central laboratory was assessed in 13 volunteers with appropriate informed consent. Blood samples were obtained and either freshly prepared and labeled or maintained at room temperature under simulated shipping conditions for 24 hours and then processed. S2 Fig shows the stability of EPCs after 24 hours at room temperature. The data shows slightly higher EPC values in 24-hour samples, with a Pearson coefficient of 0.82 and an intraclass correlation coefficient of 0.80. The intraclass correlation coefficient represents agreements between two or more evaluation methods on the same set of subjects, with 1.0 being perfect agreement.

Supplement References

1. Tracy RP, Doyle MF, Olson NC, Huber SA, Jenny NS, Sallam R, et al. T-helper type 1 bias in healthy people is associated with cytomegalovirus serology and atherosclerosis: the Multi-Ethnic Study of Atherosclerosis. Journal of the American Heart Association. 2013;2(3):e000117. Epub 2013/05/22.

2. Peichev M, Naiyer AJ, Pereira D, Zhu Z, Lane WJ, Williams M, et al. Expression of VEGFR-2 and AC133 by circulating human CD34(+) cells identifies a population of functional endothelial precursors. Blood. 2000;95(3):952-8. Epub 2000/01/29.

3. Hristov M, Erl W, Weber PC. Endothelial progenitor cells: mobilization, differentiation, and homing. Arteriosclerosis, thrombosis, and vascular biology. 2003;23(7):1185-9. Epub 2003/04/26.

4. Fraser CG, Harris EK. Generation and application of data on biological variation in clinical chemistry. Critical reviews in clinical laboratory sciences. 1989;27(5):409-37. Epub 1989/01/01.

5. Sakkinen PA, Macy EM, Callas PW, Cornell ES, Hayes TE, Kuller LH, et al. Analytical and biologic variability in measures of hemostasis, fibrinolysis, and inflammation: assessment and implications for epidemiology. American journal of epidemiology. 1999;149(3):261-7. Epub 1999/02/02.
