# Supplementary figures and images for: Endothelial progenitor cells in chronic obstructive pulmonary disease and emphysema
Source: PLoS One. 2017 Mar 14;12(3):e0173446. doi: 10.1371/journal.pone.0173446 (PMC5349667; doi:10.1371/journal.pone.0173446)

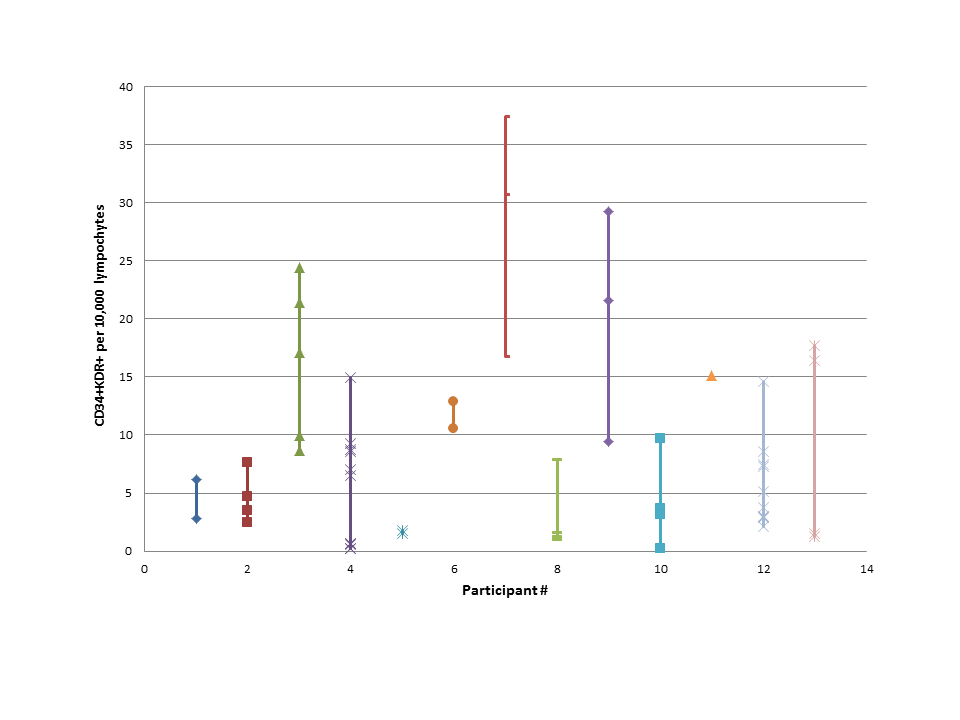

Supplement: S1 Fig — Biovariability data on 13 volunteers, measured over 18 months for CD34+KDR+ EPCs, expressed as # of CD34+KDR+ EPCs per 10,000 lymphocytes. (TIF) [file pone.0173446.s002.tif]

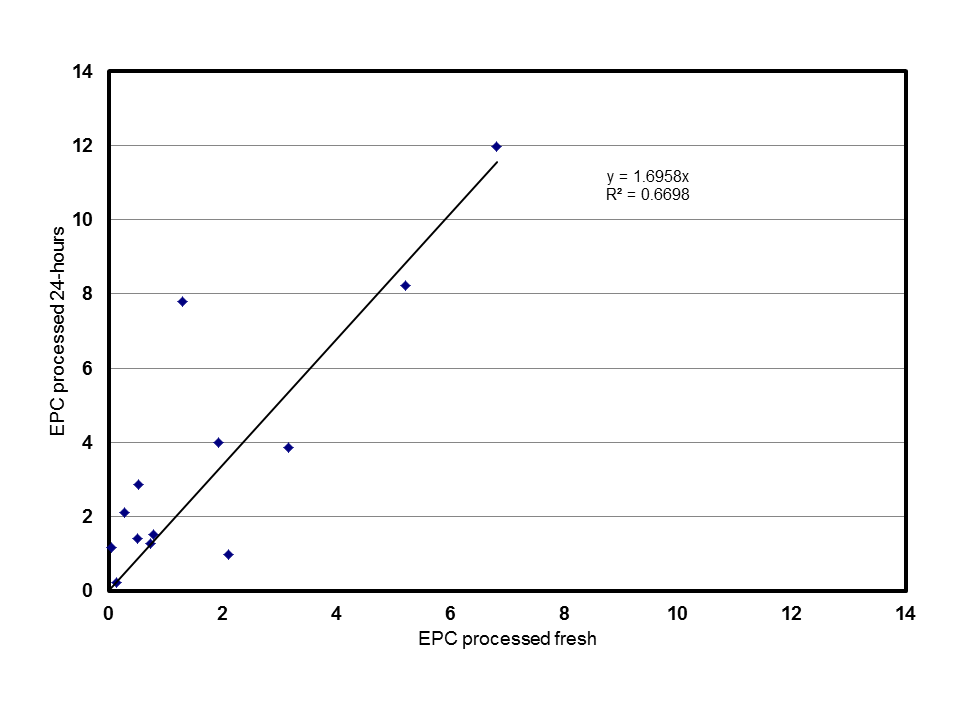

Supplement: S2 Fig — (TIF) [file pone.0173446.s003.tif]

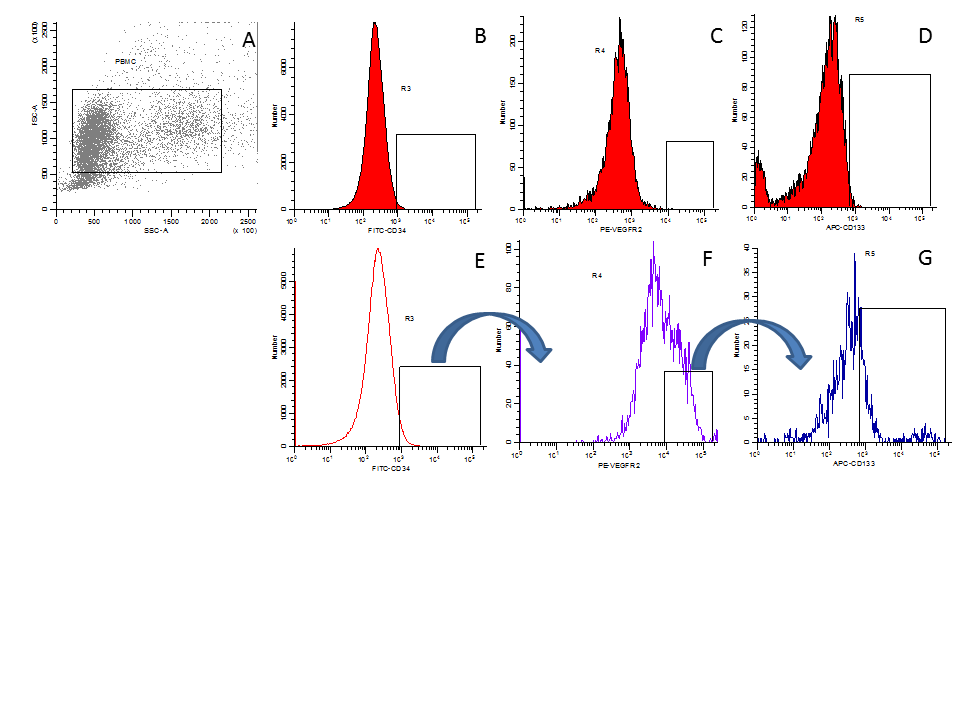

Supplement: S3 Fig — PBMCs were gated based on forward and side scatter properties. A) Forward vs. side scatter to set PBMC gate. B) FITC isotype gate of the gated PBMCs. C) PE Isotype gate of the gated PBMCs. D) APC Isotype gate of the gated PBMCs. E) FITC-CD34+ gated on PBMCs. F) PE-VEGFR2 (KDR)+ gated on the FITC-CD34+cells. G) APC-CD133+ gated on FITC-CD34+PE-VEGFR2(KDR)+ cells. Region F is used as the CD34+KDR+ EPCs and Region G is the CD34+KDR+CD133+ EPCs, both expressed as % PBMCs. (TIF) [file pone.0173446.s004.tif]

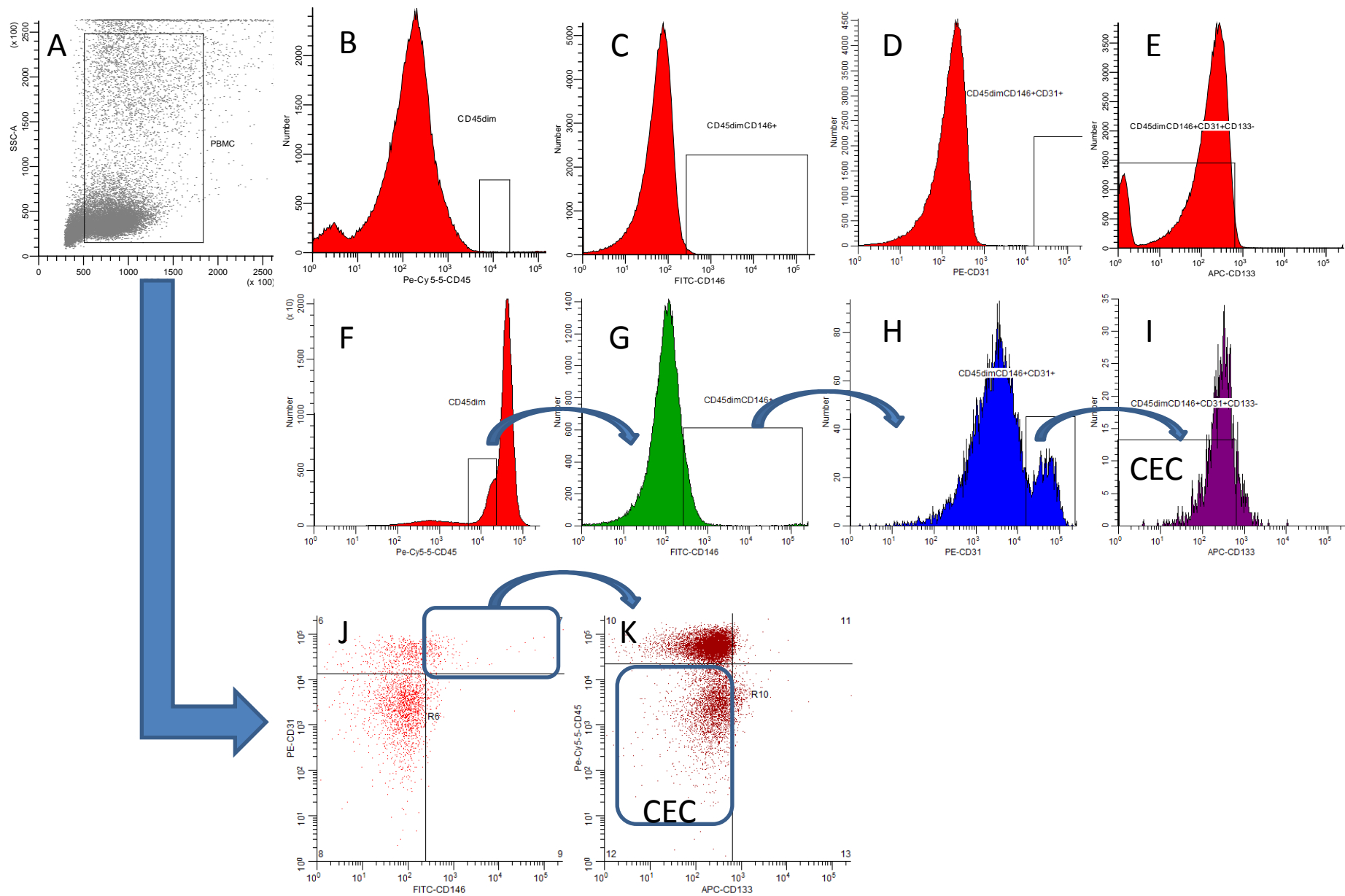

Supplement: S4 Fig — Gating strategy utilizes both quad statistics and single color histograms to determine gate placement. A) PBMCs are gated using forward and side scatter; B) PeCy5.5 Isotype gate of the gated PBMCs; C) FITC isotype gate of the gated PBMCs; D) PE Isotype of the gated PBMCs; E) APC isotype of the gated PBMCs; F) PE-Cy5.5 CD45dim gate of the gated PBMCs; G) FITC-CD146+ of the PE-Cy5.5CD45dim gate; H) PE-CD31+ of the PE-Cy5.5 CD45dim FITC-CD146+ cells; I) CD133- of the PE-Cy5.5 CD45dim FITC-CD146+PE-CD31+ cells (This CD133- population is the CECs); J) Plot of PE-CD31 vs FITC-CD146 of the gated PBMCs using quad stats; K) PeCY5.5CD45dim vs APC-CD133 plot of the PE-CD31+FITC-CD146+ cells (The PE-Cy5.5CD45dimAPC-CD133- population is the CECs). (PDF) [file pone.0173446.s005.pdf]
