# Supplementary material for: Endothelial progenitor cells in chronic obstructive pulmonary disease and emphysema
Source: PLoS One. 2017 Mar 14;12(3):e0173446. doi: 10.1371/journal.pone.0173446 (PMC5349667; doi:10.1371/journal.pone.0173446)
Supplement: S1 Table — (DOCX) [file pone.0173446.s006.docx]

S1 Table. Mean differences in endothelial progenitor cells and circulating endothelial cells according to related to pulmonary perfusion on MRI and diffusing capacity

| n=111 | **DL_CO_^a^** | **p-value** | **DL_CO_/VA^b^** | **p-value** | **Pulmonary Blood Flow^c^** | **p-value** | **Pulmonary Blood Volume^d^** | **p-value** |
| --- | --- | --- | --- | --- | --- | --- | --- | --- |
| **CD34+KDR+ as % PBMCs [x10^-3^]** | | | | | | | | |
| Model 1*  log mean difference | 0.109  (0.007, 0.211) | **0.04** | 0.276  (-0.349, 0.901) | 0.39 | -0.006  (-0.012, -0.001) | **0.04** | -0.122  (-0.212, -0.032) | **0.008** |
| Model 2†  log mean difference | 0.130  (0.030, 0.229) | **0.01** | 0.398  (-0.193, 0.989) | 0.19 | -0.006  (-0.012, -0.001) | **0.04** | -0.121  (-0.210, -0.033) | **0.007** |
| Model 3‡  log mean difference | 0.169  (0.080, 0.257) | **<0.001** | 0.718  (0.182, 1.254) | **0.009** | -0.004  (-0.012, 0.003) | 0.23 | -0.094  (-0.196, 0.008) | 0.07 |
| **CD34+KDR+CD133+ as % PBMCs [x10^-3^]** | | | | | | | | |
| Model 1,  log mean difference | 0.084  (-0.025, 0.192) | 0.13 | 0.450  (0.005, 0.896) | **0.048** | -0.003  (-0.007, 0.001) | 0.10 | -0.069  (-0.135, -0.002) | **0.03** |
| Model 2,  log mean difference | 0.087  (-0.022, 0.196) | 0.12 | 0.477  (0.047, 0.907) | **0.03** | -0.003  (-0.006, 0.001) | 0.08 | -0.062  (-0.115, -0.008) | **0.02** |
| Model 3,  log mean difference | 0.116  (0.009, 0.223) | **0.03** | 0.580  (0.136, 1.023) | **0.01** | -0.001  (-0.004, 0.003) | 0.74 | -0.020  (-0.072, 0.033) | 0.46 |
| **CEC (CD31+CD146+CD133- as % PBMCs)** | | | | | | | | |
| Model 1,  log mean difference | 0.049  (-0.025, 0.123) | 0.20 | -0.037  (-0.459, 0.385) | 0.86 | -0.009  (-0.016, -0.003) | **0.003** | -0.134  (-0.223, -0.045) | **0.003** |
| Model 2,  log mean difference | 0.058  (-0.018, 0.130) | 0.14 | 0.004  (-0.404, 0.411) | 0.99 | -0.010  (-0.016, -0.003) | **0.003** | -0.134  (-0.222, -0.045) | **0.003** |
| Model 3,  log mean difference | 0.104  (0.043, 0.165) | **<0.001** | 0.264  (-0.104, 0.633) | 0.16 | -0.006  (-0.013, 0.001) | 0.08 | -0.101  (-0.200, -0.001) | **0.048** |

^a^ per mL CO/min/mm Hg increase

^b^ per unit increase

^c^  (per ml*min^-1^*100ml^-1^ increase)

^d^ (per ml*100ml^-1^ increase)

* Model 1 adjusted for age, gender, race/ethnicity and cohort.

† Model 2 adjusted for variables in model 1 in addition to smoking status, and pack-years.

‡ Model 3 adjusted for variables in model 2 in addition to educational attainment, body mass index, height, diabetes mellitus, hypertension, oxygen saturation, white blood cell count, sleep apnea, HDL, statin use and cardiac output.
