# Supplementary material for: Endothelial progenitor cells in chronic obstructive pulmonary disease and emphysema
Source: PLoS One. 2017 Mar 14;12(3):e0173446. doi: 10.1371/journal.pone.0173446 (PMC5349667; doi:10.1371/journal.pone.0173446)
Supplement: S2 Table — (DOCX) [file pone.0173446.s007.docx]

S2 Table. Association between EPCs, CECs and air trapping

|  | **Change in Percent below -856 HU** | **p-value** |
| --- | --- | --- |
| **CD34+KDR+ as % PBMCs [x10^-3^]** | |  |
| Model 1, log mean difference | -0.014 | 0.19 |
| Model 2, log mean difference | -0.016 | 0.11 |
| Model 3, log mean difference | -0.022 | **0.01** |
| **CD34+KDR+CD133+ as % PBMCs [x10^-3^]** | |  |
| Model 1, log mean difference | -0.004 | 0.62 |
| Model 2, log mean difference | -0.004 | 0.58 |
| Model 3, log mean difference | 0.002 | 0.72 |
| **CEC (CD31+CD146+CD133-) as % PBMCs [x10^-3^]** | |  |
| Model 1, log mean difference | -0.005 | 0.58 |
| Model 2, log mean difference | -0.005 | 0.52 |
| Model 3, log mean difference | -0.012 | 0.07 |
| **CD34 as % PBMCs [x10^-3^]** |  |  |
| Model 1, log mean difference | -0.007 | 0.44 |
| Model 2, log mean difference | -0.008 | 0.39 |
| Model 3, log mean difference | -0.013 | **0.047** |

Model 1 adjusted for age, gender, race/ethnicity and cohort.

Model 2 adjusted for variables in model 1 in addition to smoking status, and pack-years.

Model 3 adjusted for variables in model 2 in addition to educational attainment, body mass index, height, diabetes mellitus, hypertension, oxygen saturation, white blood cell count, HDL, sleep apnea and statin use.
